# Applying the model of diffusion of innovations to understand facilitators for the implementation of maternal and neonatal health programmes in rural Uganda

**DOI:** 10.1186/s12992-019-0483-9

**Published:** 2019-06-13

**Authors:** Ligia Paina, Gertrude Namazzi, Moses Tetui, Chrispus Mayora, Rornald Muhumuza Kananura, Suzanne N. Kiwanuka, Peter Waiswa, Aloysius Mutebi, Elizabeth Ekirapa-Kiracho

**Affiliations:** 1 Health Systems Program, Department of International Health, Johns Hopkins University Bloomberg School of Public Health, Baltimore, MD USA; 2 Department of Health Policy, Planning and Management, Makerere University School of Public Health, New Mulago Complex, Kampala, Uganda; 3 Epidemiology and Global Health Unit, Department of Public Health and Clinical Medicine, Umeå University, 901 87 Umeå, Sweden

**Keywords:** Uganda, Innovation, Maternal health, Health systems research, Vouchers, Diffusion

## Abstract

In Uganda, more than 336 out of every 100,000 women die annually during childbirth. Pregnant women, particularly in rural areas, often lack the financial resources and means to access health facilities in a timely manner for quality antenatal, delivery, and post-natal services. For nearly the past decade, the Makerere University School of Public Health researchers, through various projects, have been spearheading innovative interventions, embedded in implementation research, to reduce barriers to access to care. In this paper, we describe two of projects that were initially conceived to tackle the financial barriers to access to care – through a voucher program in the community - on the demand side - and a series of health systems strengthening activities at the district and facility level - on the supply side. Over time, the projects diverged in the content of the intervention and the modality in which they were implemented, providing an opportunity for reflection on innovation and scaling up. In this short report, we used an adaptation of Greenhalgh’s Model of Diffusion to reflect on these projects’ approaches to implementing innovative interventions, with the ultimate goal of reducing maternal and neonatal mortality in rural Uganda. We found that the adapted model of diffusion of innovations facilitated the emergence of insights on barriers and facilitators to the implementation of health systems interventions. Health systems research projects would benefit from analyses beyond the implementation period, in order to better understand how adoption and diffusion happen, or not, over time, after the external catalyst departs.

**Electronic supplementary material:**

The online version of this article (10.1186/s12992-019-0483-9) contains supplementary material, which is available to authorized users.

## Background

Maternal and newborn mortality is an important issue in Uganda. Though it remains high, maternal mortality decreased by a quarter between 2011 and 2017, from 438 to 336 women per 100,000 live births [1, 2]. In contrast, newborn mortality, of 27 newborns per 1000 live births dying annually, has been stagnant for the last decade, and has been especially persistent in rural areas [1, 2]. Low coverage of skilled birth attendance and emergency obstetric care, inadequate birth spacing and poor postnatal care represent the principal underlying factors responsible for the high mortality figs. [3–5]. For example, one in four pregnant women does not deliver under the care of a skilled birth attendant [1, 2]. Demand side barriers include the cost of services from informal fees or supplies that patients have to buy on their own, cost of transport, difficult terrains, lack of knowledge about obstetric danger signs, and misconceptions about pregnancy, birth, and newborn care rooted in cultural beliefs [4–8]. The cost of services is an issue despite the fact that, in Uganda, maternal and newborn health services are supposed to be provided for free in all health facilities, with the exception of hospital private wings [9]. Supply side barriers include inadequate numbers of skilled health workers, poor health worker attitudes due to low motivation and remuneration, poor performance management and inadequate supplies and equipment required for service delivery [5, 7, 10, 11]. In response to the above challenges, Uganda sharpened its priorities for reproductive, maternal, newborn, and child health in order to achieve the maximum and quickest gains for mothers and children [12] According to this plan, Uganda is undertaking several strategic shifts with increased focus on increasing access to services in underserved geographical areas and to populations with a high burden, scaling up and measuring coverage of high impact interventions, strengthening health system functionality and promoting mutual accountability [12].

Researchers from the Makerere University School of Public Health (MakSPH) have also been testing various packages of interventions to tackle these barriers. A series of projects that MakSPH researchers have implemented in the past decade are of particular interest to this short report. Specifically, we focus on the Maternal and Neonatal Implementation for Equitable Systems’ (MANIFEST) and the Maternal and Newborn Care Practices Study’s (MANEST) projects, which were implemented in rural Uganda. At the time they were introduced, they represented unique combinations of supply and demand-side interventions, which sought to use implementation research to overcome financial and non-financial barriers to health service utilization that have previously hampered the institutionalization of evidence-based maternal and newborn health interventions into local systems.

While many refer to innovations as products (i.e. drugs, diagnostics, new technologies), we apply the concept to “projects to overcome resource constraints” [13], in this case to overcome persistent barriers to access to services and care for mothers and newborns. Therefore, for the purpose of this short report, we consider the two projects and the implementation of their packages of interventions as the innovations of interest. Examining the implementation of the two projects allows us to reflect on lessons learned from introducing new interventions into a system and understanding barriers and facilitators to implementation and implications for diffusion and scale-up.

In this short report, we aim to reflect on the lessons learnt from these two projects, particularly focusing on the MANEST and MANIFEST implementation processes, by adopting an adaptation of the Greenhalgh’s Model of Diffusion of Innovation [14]. We are not able to discuss whether or not the innovations were diffused, but rather we consider the barriers and facilitators for diffusion, which are broadly important in the management of complex issues, such as increasing access to quality maternal and newborn care in resource limited settings. We conclude with reflections on the potential of the project’s diffusion and the role of understanding innovation for health systems research implementation. While the main research findings from the MANEST and MANIFEST projects are published elsewhere [15–18], this short report describes the teams’ reflections on barriers and facilitators to the implementation of the interventions, including a descriptive analysis framework for conceptualizing the projects as innovations.

### History and structure of the MANEST and MANIFEST projects

In 2012, MakSPH obtained additional funding from FHS and Comic Relief, as well as the World Health Organization to build on the Safe Deliveries and UNEST programs and catalyze further gains in access to quality maternal and newborn health services in rural Uganda. From 2012 to 2015 they used this funding to implement the MANEST and MANIFEST projects.

MakSPH had implemented the Safe Deliveries [19] and the Uganda Newborn Study (UNEST) [20] projects between 2009 and 2011. Both projects aimed to increase access to quality maternal and newborn health service delivery in rural areas, but which had slightly different intervention packages and implementation strategies. Safe Deliveries, funded by the UK Department for International Development through the Future Health Systems Research Programme Consortium (FHS RPC), provided free transport and service vouchers to pregnant women to reduce the cost of seeking care, as well as to enhance antenatal care, delivery and postnatal care service uptake. UNEST, funded by Saving Newborn Lives of Save the Children USA/Uganda through a grant from the Bill & Melinda Gates Foundation, supported community health workers to provide health education during home visits to improve maternal and newborn care practices, as well as basic equipment and supplies to participating facilities. Both projects strengthened facility capacity by conducting refresher trainings for health workers on maternal and newborn health topics and by providing basic equipment and supplies, as well as supportive supervision to the participating facilities. At the end of the implementation period, these projects achieved increased utilization of maternal and newborn services and improvements in some newborn care practices within the areas of intervention [21]. However, common to pilot projects, neither of them had the financial resources, from the government of Uganda or otherwise, necessary to scale up the projects immediately. The funding from WHO, Comic relief and FHS therefore provided an opportunity for the Makerere team to continue implementation through the MANIFEST and MANEST projects.

Although MANEST and MANIFEST were implemented separately, the research team initially designed them as one project. This project intended covering 6 districts and spearheading the implementation of the Ministry of Health’s Village Health Team strategy, strengthening the health system through health worker training, support supervision, and performance bonuses, as well as providing mothers with transport vouchers for safe delivery. While the World Health Organization (WHO) and FHS provided funding to start this project, a third funder – Comic Relief made some additional amendments during the design phase, emphasizing that they would not be able to support transport vouchers or performance bonuses directly. In order to meet Comic Relief’s amendments, the project was split into two independent arms. MANEST, supported by WHO and FHS, kept the initial design and was launched first, starting with a formative research phase. MANIFEST, supported by FHS and Comic Relief was developed based on formative research which included broad consultations with communities, district authorities, health workers, policy makers, and the requirement by the funder Comic Relief to design a project which was embedded in existing structures and driven by communities in order to enhance sustainability [22]. Both MANEST and MANIFEST shared research team members. EEK, GN, AM, and PW were a part of both MANEST and MANIFEST. CM, RKM, MT, and SNK were only a part of the MANIFEST project. Sharing research team members facilitated learning and sharing between the two projects. LP was not directly involved in the design or implementation of either project, but backstopped the Uganda FHS team during the second half of the Future Health Systems project.

Table 1 summarizes the structural elements of the two projects, highlighting the main differences and similarities. For example, MANEST had a much smaller budget than MANIFEST, limiting the flexibility of MANEST’s design and the ability to incorporate participatory elements, such as district meetings. MANIFEST was implemented on a slightly larger scale than MANEST – MANIFEST worked in 3 health sub-districts, while MANEST worked in 2 health sub-districts and a demographic surveillance site. With the additional funding, MANIFEST adopted a participatory action research (PAR) approach, meaning that the community and other key stakeholders were engaged and consulted throughout the project implementation. [23–25].

**Table 1:** Summary of key project characteristics

Project characteristics	MANEST	MANIFEST
Budget	$700,000	$2,000,000
Duration	3 years	3 years
Funding sources	FHS/DFID – for implementation WHO – for implementation	Comic Relief – for implementation FHS/DFID for technical support
Study design	Quasi-experimental design	Quasi-experimental design + Participatory Action Research
Area of intervention	2 intervention health sub-districts 1 control health sub district	3 intervention health sub-districts 3 control health sub districts
Population	1.0 Million	1.07 Million
Model of diffusion	Closed, replication, not scale-up	Open, flexibility in design; but not scale-up per se

Both MANEST and MANIFEST included support supervision and mentorship, training for health workers, sensitizing transporters, and some level of community engagement. However, they used slightly different implementation strategies. For example, for support supervision of community health workers, MANEST used Super Village Health Teams (VHTs) (an Super VHT is the leader of all Village Health Teams’ in the parish) for support supervision of community health workers, whereas the MANIFEST team used directly observed supervision by health workers. On community mobilization, MANIFEST implemented community dialogues, which were initially intended as a PAR component, but later also served as an important sensitization approach. MANEST, did not hold regular community meetings for sensitization beyond the launch of the project, but trained community health workers to engage with the community during regular events, such as burials and church-events.

Some of the intervention components were unique to each project. Per the donor requirements mentioned in the background, only MANEST featured transport vouchers and performance bonuses for health staff, while only MANIFEST featured a community mobilization component as part of the intervention. MANIFEST aimed to promote a saving culture within the community for households to prepare for future pregnancy, and birth-related needs, including local transport and buying medical supplies, and other health emergencies [26]. This, the project did, though encouraging households and women to join either already existing savings associations or to form their own saving groups to save money [26]. Table 2 synthesizes the similarities and differences between the two projects in terms of the intervention components that they adopted.

**Table 2:** Summary of similarities and differences among intervention components

Intervention components	Manest	Manifest
Support supervision and mentorship	Yes	Yes, different implementation strategy Quarterly support supervision with district health team and quarterly mentoring with external and internal mentors.
Performance incentives	Financial incentives	Health worker and facility performance recognition and other non-financial incentives
Sensitization of transporters	Yes	Yes
HW Training	Yes	Yes
Transport vouchers	Yes, in 1 district out of 3	No transport vouchers
Saving groups	No	Yes
Community health worker engagement strategy	Encouraging VHT to use any opportunity they have when they meet people to do sensitization such as at burrials and churches. House to house registration and visits	Community dialogue meetings House to house registration and visits

### Conceptual framework

To aid our reflection, we adapted Greenhalgh’s framework for the diffusion of innovations [14]. The framework was developed over a series of reflection meetings (May 2015, June 2015, and September 2016) during which the authors, who also participated in the implementation of the research projects, identified the innovation concepts most relevant to understanding the two projects and how they evolved over time, as well as key project documents to be reviewed.

**Fig. 1:**
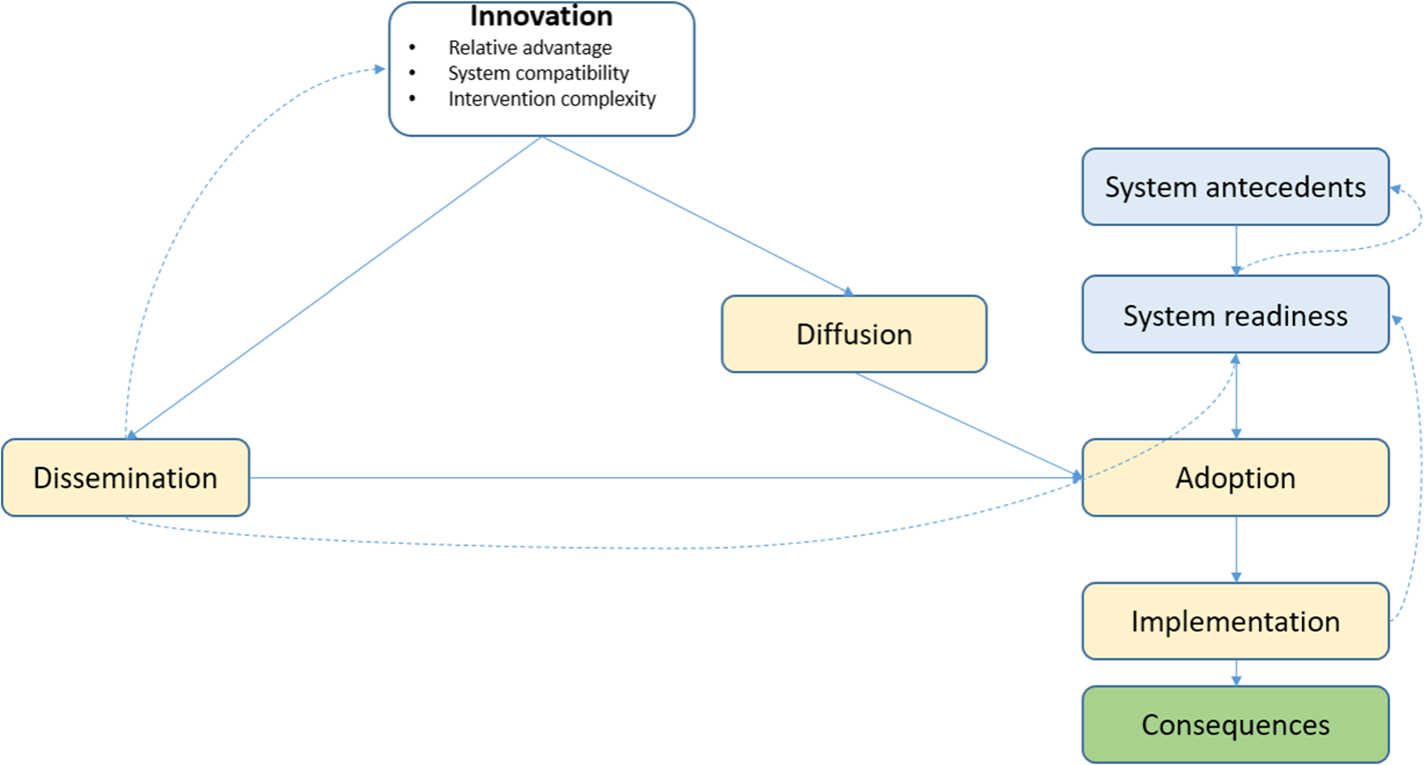
Conceptual framework – Model of Diffusion, adapted from Greenhalgh et al. [17]

Greenhalgh and colleagues intended the Model of Diffusion as a “memory aide” to facilitate and guide a process of critical thinking about the complex aspects of the innovation and the system in which it is introduced, and how these might interact as adoption occurs [14]. For the purpose of this paper, the authors selected the subset of Model of Diffusion concepts that would best describe aspects of MANEST and MANIFEST implementation (see Fig. 1). Specifically, the teams selected the concepts that related to either the stated intent of the projects or to themes that emerged through the implementation and the reflection processes. The figure understates the non-linear nature of the processes of implementation discussed here, but the dotted arrows begin to address this and feedback between the various concepts is elaborated further.

The innovation itself is represented in the top part of the framework. MANEST and MANIFEST, the two innovations of interest, will be compared and contrasted along the following: relative advantage, compatibility, complexity, reinvention, risk, task issues, and knowledge required to use it [14]. On the right hand side of the conceptual framework, we highlight the factors related to the adoption of the innovation by individuals, which are likely to be very similar between the two projects. On the left hand side, we highlight the system components which facilitate the spread of innovation – both in terms of the system antecedents (i.e. structural factors that had to be in place), as well as system readiness factors (i.e. existing tension or pressure for change, the innovation-system fit, and dedicated time and resources).

Framing the two projects in terms of these characteristics will also facilitate the reflection upon the pathways through which the innovations could be adopted. Greenhalgh et al. suggest that “the various influences that help spread the innovation can be thought of as lying on a continuum between pure diffusion ([ …] unplanned, informal, decentralized, and largely horizontal and mediated by peers) and active dissemination ([ …] planned, formal, often centralized, and likely to occur through more vertical hierarchies)” [14]. Our framework does not fully elaborate the proposed continuum, but presumes that an innovation can be adopted through either dissemination or diffusion, or both. By design, MANEST and MANIFEST intended scale-up or adoption of the project components to happen primarily through dissemination – strengthening, on the one hand, the capacity of local structures and, on the other, especially for MANIFEST, local leader and community engagement. While the existing data does not allow us to examine scale-up and adoption in practice, nevertheless, we thought it would be useful to explore factors that could facilitate diffusion as well.

Finally, the framework recognizes the importance of implementation processes in the spread of innovations, acknowledging that different implementation factors and approaches may have different consequences. It is beyond the scope of this paper to speculate about the performance of the two projects and whether different project approaches led to different outcomes. Nevertheless, the framework includes a discussion of consequences, to highlight that different strategies for spreading interventions might have different outcomes, which are worth evaluating when possible.

The information from which the reflections below arise was collected based on a review of project documents and publications, as well as a couple of reflection meetings and authors’ contributions to the first draft of the manuscript. In order to facilitate brainstorming sessions on the details related to project design and implementation, EEK walked the team through a semi-structured guide, which was developed based on the framework described above (See Additional file 1). Additionally, LP used the proposed framework to guide the review and data extraction of information from project proposals and work plans relevant to the MANEST and MANIFEST projects. The extracted information was discussed during a final reflection meeting, facilitated by EEK in September 2016. The reflections shared below represent the two project team’s perceptions, as we did not have the opportunity to triangulate these with any related stakeholders. Reflections on MANEST and MANIFEST projects as innovations.

#### Innovation-related project characteristics

Retrospectively, MANEST’s implementation was premised on a closed or limited model of innovation, one that was focused on replication of interventions, with little flexibility for adaptation [27]. The Maternal and Neonatal Implementation for Equitable Systems’ (MANIFEST) implementation followed an open model of innovation, one with the flexibility to adapt over time in response to contextual stimuli and work to unlock community capabilities [27]. From the teams’ perspectives and based on consultations with local stakeholders, both MANEST and MANIFEST proposed interventions that had a relative advantage and were relatively compatible with the existing system – effectively, the interventions were perceived to address important barriers to access and quality of care, and they were seen as complementary to other services offered. Team members from both projects expected that recipient communities would be able to see a direct benefit from the interventions and therefore, demand health care. For example, community members appreciated the importance of home visits by Village Health Team members. District health teams appreciated project support to deliver on their mandate. The compatibility of interventions with existing structures was probably the most important facilitator to implementation and a key indicator of system readiness. By working through existing health system structures, both projects enhanced current, on-going processes rather than create new ones. Furthermore, through PAR, MANIFEST was able to tailor the intervention to existing local structures more so than MANEST. For example, existing savings groups in intervention communities were engaged as savings schemes for maternal, newborn, and child health care services and linked to local transporters so as to increase the availability of both cash for meeting maternal and newborn needs and transport to the facility. Despite the interventions’ presumed compatibility, some skepticism arose, particularly from private transporters, in response to some of the uncertainty or risk that they were expected to take on as part of MANIFEST activities. For example, transporters declined to participate in the intervention when they perceived that there was a chance that they might not be paid for taking women to and from the health facility in a timely fashion, due to contracting delays. Transporters who were linked to savings groups or were part of the savings groups themselves were more comfortable with these arrangements.

Intervention design and evolution also played a role in the extent to which perceived risk could be minimized to enhance the participation of the various actors. For instance, through MANEST, the project could only contribute to one-way transport to health facilities, although transporters were often expected to provide round-trip services. Moreover to reduce the risk of loss of project funds, the transporters were paid initially by cash and eventually through mobile money accounts, based on the proliferation of mobile money infrastructure across Uganda. The other advantage of mobile money was that it could be paid anywhere and anytime. However transporters who did not have active mobile money accounts or were not familiar with the technology were at a disadvantage.

In terms of innovation complexity, MANIFEST’s intervention package was more complex than MANEST’s, which, for some intervention components, resulted in more adaptation, longer learning, and slower implementation. Specifically, MANIFEST attempted to influence norms around the community’s role in maternal and newborn health. It aimed to shift the role of community members from being merely recipients of services to being active contributors to service delivery – specifically in terms of mobilizing funds and transportation for women who needed it. MANIFEST also attempted to change district and health facility-level norms by introducing the use of local mentors. The mentorship approach was new, complex, and difficult for district and facility stakeholders to take up, especially in the first year of the project. The implementation team therefore decided, in tandem with district authorities to use the first year as a learning period to refine the mentorship process. This delayed the scaling up of the implementation to other facilities in the district.

While both projects grappled with the complexity of some of the intervention components, the teams noted that some components were more readily adopted by target districts and communities than others. For example, for MANEST, implementing through VHTs was easy to relate to and other projects were also working through these same channels. However, structural changes, such as the addition of a newborn resuscitation corner or kangaroo mother care in health facilities were slower to be taken up by the community. Overall, for both projects, it seemed as whenever the community appreciated the value of a component or saw practical benefits to having it (e.g. making savings, birth preparedness), they were more willing to take them up, even if such interventions were relatively complex to implement.

#### System antecedents and readiness

The Ministry of Health’s Village Health Team policy, which preceded the two projects, was the most important system antecedent that influenced their implementation. In order to overcome some of these challenges, the Ministry of Health had introduced a strategy for community mobilization and awareness creation through community health workers – in Uganda, called Village Health Teams [28].

The Village Health Team policy had taken several years to be implemented, though a framework was in place. The implementation of the Ministry’s Village Health Team policy was slow and plagued with numerous challenges, raising demand for arrangements that could facilitate formal engagement of community health workers in service delivery, particularly for maternal and newborn health.

Each village is expected to have five community health workers who comprise the village health team. At the start of both the MANEST and MANIFEST projects, Village Health Teams had been selected only in a few areas across the country and supported by external funding and technical assistance and struggled where local structures to integrate community health workers within the broader health system were not in place. For instance, initial debates related to the implementation of Village Health Teams centered on whether and how to remunerate community health workers and how to set up a system for supervision. MANEST and MANIFEST therefore concentrated on these and tested different approaches to addressing these issues. In the process, both projects experienced the challenge of adequately involving higher-level stakeholders to use the research for decision-making. The use of data for decision-making trickled all the way down to the facility level, where health facilities began collecting data, such as performance reviews, which was useful for daily decision-making.

The implementation of MANEST and MANIFEST predecessor projects (i.e. UNEST and Safe Deliveries) [29, 30], coupled with increasing visibility of newborn health issues on the political agenda at the national and district levels contributed to system readiness and stakeholder buy-in. Additionally, there was a reasonable understanding and consensus among stakeholders and national and district levels on the key barriers that had to be overcome in order to promote safe deliveries and maternal and newborn survival. Furthermore, the two projects, and especially MANIFEST through PAR, provided support and advocated for intervening at multiple levels in the system. Through this advocacy, as well as the system’s readiness, the projects were able to understand how to better leverage the relatively few resources they had available to meet intervention goals and how to navigate and engage various stakeholders to facilitate implementation. Although they did not use PAR, the precursor projects [29, 30], as well as MANEST regularly invited stakeholders to project events in order to share with them the emerging findings of the research and implementation. For example, through meetings with the leaders of transporter associations, the Safe Deliveries project had learned to be responsive to the way in which transporters organized themselves and how best to engage stakeholders in implementation. MANIFEST was also designed to explore community resourcefulness toward maternal health in order to mitigate the unsustainable transport cost challenge experienced during the Safe Deliveries project.

#### Reflections on implementation

Both project teams embedded, through their standard operating procedures, opportunities to reflect on implementation and on communicating about the intervention with key stakeholders. These procedures were modified in light of findings from the formative research and after the intervention was piloted. During implementation, the MANEST intervention did not change very much. The quarterly meetings that the research team organized with implementers were used for monitoring the process and for providing refresher trainings rather than fostering discussions around adapting the intervention. MANIFEST, on the other hand, organized regular meetings with stakeholders at various levels in the health system, to monitor the interventions, but also engaged various stakeholders in decision-making around project implementation [24, 31]. Using PAR, quarterly review meetings among research team members, sub-county officials, and the intervention’s advisory committee, as well as broader stakeholder meetings and workshops at the national level served as a forum for identifying solutions to implementation challenges and understanding the context. These meetings also provided opportunities for the project to disseminate information to key stakeholders, and thereby facilitated adoption. Each project’s approach to stakeholder engagement approach depended primarily on the flexibility of funding arrangements. MANIFEST’s funding sources allowed for flexible adaptation of the intervention and therefore led to more stakeholder engagement. MANEST’s project design and funding on the other hand were based on a fixed design, and therefore engaged stakeholders mostly in the dissemination of information.

A key barrier to implementation, and ultimately to adoption and diffusion of both projects was tied to the system readiness and system antecedents mentioned above. Where structures were in place, implementation was facilitated. However, where structures, guidelines and standard operating procedures were missing or dysfunctional, the implementation was slower and adoption was delayed. For example, while community development officers were part of the government structure and they were expected to support saving groups, in reality they lacked the capacity to perform this role both in terms of the skills and financial resources required. Furthermore, broader health systems problems such as drug shortages, poor health worker attitudes and inadequacies also constrained implementation, in addition to challenges such as long distances between households and communities, poor roads and infrastructure. Finally, deeply entrenched social norms and customs around newborn care (i.e. bathing babies right after birth, applying substances to the umbilical cord) created social cultural barriers within the implementation environment.

#### Reflections on adoption and diffusion

The teams did not objectively evaluate whether the projects encouraged diffusion, nor did they evaluate whether diffusion contributed to contamination for purposes of evaluating their control areas. The MANIFEST project observed that some of their intervention components spread unexpectedly beyond the intervention areas. For example, radio spots, through which community members were mobilized and encouraged to join savings groups for maternal and newborn care, were heard outside of the intervention area and the end line evaluation also showed a slight increase in savings groups even in the control area. However, it is unclear to what extent this unexpected spread actually led to increased knowledge about savings for maternal and newborn care or whether it facilitated the acceptability of using savings groups for this purpose. The projects did not consider limiting diffusion, per se. On the contrary, other implementing partners were invited to regular project meetings and it is possible that they could have taken some ideas and transferred or adapted them for their own projects. But, the extent to which this happened has not been purposefully documented. The practice of holding review meetings in one of the intervention districts for MANIFEST has been spread beyond the intervention areas to the entire district. However, it cannot be determined whether this diffusion happened as a result of the MANIFEST project or as a result of some other trend (e.g. some districts might have been planning to incorporate these in any case, the district health officer was a champion). Based on what MANIFEST researchers observed, one of districts under their project has taken up the recognition of health workers (through award of certificates and related motivations) with non-financial incentives, albeit with challenges of performance measurement. In other cases, project team members have observed potential barriers to diffusion, related to system readiness, resource availability and cultural constraints. For example, some of the MANIFEST districts wanted to scale-up mentorship but the districts lacked the resources for transporting mentorship teams around more facilities and to hire enough mentors to cover a sufficient number of facilities. Nonetheless, the MANIFEST team designed mentoring of health workers as a cascade by strengthening the capacity of local mentors to continue the practice to other health workers within the districts [32]. Team members also proposed that, based on their observations, the implementation of transport vouchers through the Safe Deliveries and MANEST projects in Eastern Uganda could have influenced the World Bank-supported voucher pilot in Western Uganda.

Local leadership at the district, facility and community level was a critical facilitator for both adoption and diffusion, particularly in the MANIFEST project. For example, wherever an active savings group manager was found, it was easier and more fruitful to liaise with and gain the trust of transporters. Strong leadership also allowed for better adaptation to local conditions. For example, initially, the project required savings groups to sign formal agreements with enlisted private transporters, limiting women to only choose transporters from this list. However, this proved problematic in practice and savings groups’ leaders started arranging informal agreements with transporters, thereby creating more flexibility for mothers. As a result of this revised approach women could look for any transporter whenever they needed to go to the hospital and were not just limited to those on the list. The savings groups with strong leadership also provided flexibility in terms of the types of services they would cover. For example, they would not limit membership to only pregnant women, but also allowed others, such as women with disabled children, or anything else that would suit the local population’s needs. While strong leadership has been shown to be important for diffusion, adaptation, and adoption, any changes in leadership can be disruptive. For example, MANIFEST recently saw an election in the districts they were working in, at the end of the project. It is possible that the newly elected leaders who lack enthusiasm for the interventions might not prioritize them, leading to inadvertent discontinuity of the intervention.

The transfer of health workers, a government policy over which the two projects did not have control, represented both a barrier and a potential opportunity for the two interventions. On the one hand, the transfer of an individual whose capacity was built by the project represented the loss of a champion. On the other hand, the person who moved to another area would be able to use their newly acquired skills and approaches and diffuse the innovation further. In the case of MANEST and MANIFEST, an enabling environment was key for the success of the intervention, and therefore, perhaps a single individual would not be able to achieve similar goals as in the former intervention area. Nonetheless, health workers who were exposed to the projects’ activities, but were subsequently transferred could be an interesting subject for further research on the spread of innovation.

Cross-project collaboration was an important means through which ideas diffused over time and also from one team to another. For example, lessons drawn from MANEST and predecessor projects were transferred to the design of the MANIFEST project, which started a bit later. While directly observed supervision of VHT by health workers for VHTs was useful and effective, the cost was too high when looking to scale-up this element of the intervention through MANIFEST. Similarly, super VHTs – an approach through which one of the VHTs was appointed to supervise a group of VHTs – and the development of VHT associations was picked up by MANIFEST from MANEST. The cross-project collaboration was most easily facilitated by the fact that the two projects shared many of the staff.

### Key lessons learned from the design and implementation of MANEST and MANIFEST

As we conclude, we summarize the key lessons learned from the design and implementation of the MANEST and MANIFEST projects and the characteristics that could facilitate spread – both in terms of adoption and diffusion.

Several projects’ characteristics that would be conducive to diffusion came through the teams’ reflections. Both project teams appreciated the PAR design through which MANIFEST was implemented, which allowed for the *active engagement of district officers and other stakeholders throughout implementation*. Both project teams also appreciated the importance of *on-going active monitoring and dissemination* of the findings to various actors in the system (at national, districts, sub-county levels, as well as through community meetings and various events hosted by the project – e.g. health worker symposia, regular stakeholder meetings) in order to ensure and maintain stakeholder buy-in. The *engagement of community members* facilitated changing their perceptions about the role of mobilizing community resources and led to more community members focusing and contributing their resources towards key health events. This was evident through savings groups – through which community resources, rather than project resources were invested in ensuring timely care for pregnant women and newborns. Furthermore, for both MANEST and MANIFEST, the greater the *relevance of the intervention* to the community, the easier it was to facilitate its adoption.

One of the core strengths of the two projects was the *strong alignment and compatibility between the characteristics of the interventions they introduced and system antecedents and readiness*. In both projects, the interventions were designed to be rolled out through existing processes, making them more compatible with the existing system. The PAR design employed by MANIFEST allowed stakeholder engagement before the start of implementation and on an on-going basis, giving the project an opportunity to be responsive to changes in the environment and to emerging phenomena. In practice, both projects teams remained with unanswered questions about the importance of compatibility – as they found that in some cases deeply entrenched social norms and customs in all of their communities as well as dysfunctional systems posed barriers to implementation, adoption, and diffusion despite the intervention itself being compatible with the expected system standards. The compatibility of the projects in practice was not evaluated directly, but would be a helpful addition to similar project evaluations. Although, in our conceptual framework, we specified the project or intervention characteristics as separate from the system antecedents and readiness, our analysis of the two projects presented in this paper highlights the importance of the innovation having embedded linkages with the system.

Finally, *local leadership was key for implementing the intervention* and, especially the MANIFEST project team, recognized the importance of strong leadership in the potential adaptation and scale up of the various activities implemented. *Early and constant involvement of leaders at all levels*, through both active engagement in decision-making about the project and dissemination of project findings was critical. Fostering relationships with key stakeholders and reaching the point of facilitating adoption does seem to benefit from long-term engagement. Any changes in this leadership can compromise the outcomes of the intervention due to the loss of champions. The Makerere University School of Public Health teams had been working in this area for many years, first through the UNEST and Safe Deliveries Projects, and then through MANEST and MANIFEST. The long-term engagement was necessary for cultivating trust and for allowing the research teams to fully understand the implementation context in which their activities would be implemented. Furthermore, it provided them with frequent opportunities to engage with stakeholders at multiple levels, to develop their capacity, and to develop the team’s internal capacity to understand the different intervention designs and their inherent benefits and challenges, opportunities and facilitators of future adoption, scale-up and sustainability. Nevertheless, the extent to which the intervention has been scaled-up or diffused is not well known. Further collaboration with private sector and non-state actors and implementing partners (such as NGOs) would be worthwhile, as they might have resources available in the short term to advance some of these interventions.

Overall, the findings in this report suggest that more research should be carried out to systematically understand the influence of funding arrangements and stipulations on research design, how to facilitate collaboration and sharing across related projects if they are implemented by the same team, and carrying out post-hoc evaluations to understand whether and how project elements diffuse or scale-up over time. Further knowledge on any of these would help implementers and researchers adapt their programmatic strategies so as to encourage the diffusion of innovations in dynamic health systems. In the case of MANIFEST, more flexible funding allowed for greater stakeholder engagement and adaptation of the intervention over time. The MakSPH research team’s long term engagement in maternal and newborn health and implementation research in the rural districts where the MANEST and MANIFEST projects were located helped to better understand system readiness in relation to introducing various intervention components. It also created an environment through which the two project teams extensively shared project information. These considerations could be relevant in how to organize local research and implementation responses in order to stimulate progress towards the Sustainable Development Goals, particularly as related to health and well-being.

Our lessons learned are limited by the fact that the implementation of the two research projects has recently ended, and therefore it is difficult to draw final conclusions about scale-up, spread, and diffusion of innovations. Building on the work of their predecessor projects, MANEST and MANIFEST have catalyzed the adoption of certain interventions, which the community, district, and national level stakeholders found acceptable. By examining the projects retrospectively, we can draw some lessons about the historic and contextual factors that facilitated the inception and implementation of the MANEST and MANIFEST projects. We are limited in any future-looking/prospective analysis, as the diffusion of the current interventions is yet to reach its full potential.

Future research should explore what happens in the implementation areas in the short and medium term, in order to determine which of the intervention components have been adopted and spread beyond intervention period and areas. Post-hoc evaluations are not the norm in health systems research – nor in Uganda, nor globally. However, in environments where activities are time-limited by nature and funding, learning from post-hoc evaluations would further build the evidence base about whether and how projects or specific interventions are disseminated, diffused, and/or adopted in the areas of implementation and beyond. This type of analysis would explore the stakeholders that have been involved in carrying out any of these activities, including other implementing partners, beyond the public sector stakeholders who were primarily involved in the MANEST and MANIFEST projects. Future projects should actively consider how the intervention might be adopted and/or diffused from the intervention design phase. Research should also explore the extent to which health workers or leaders transfer to other locales are able to diffuse these interventions within their new contexts. Finally, cross—project collaboration probably happens often, though probably only informally in many institutions. More learning about benefits and drawbacks of intra-institutional cross-project collaborations, as well as the benefits and drawbacks of having similar concurrent projects should be further studied, exploring synergies as well as risks for duplication. More broadly, the reflection summarized in this paper draws attention to the value of using implementation research to understand complex projects, which introduce multiple innovative interventions or practices in a particular area. The development of a heuristic, possibly based on an adapted model of diffusion could facilitate learning and synthesis for advancing insights into factors the facilitate diffusion and implementation of complex interventions, as well as cross-project collaboration. In the MANEST and MANIFEST case, the collaboration seemed to have promoted information sharing and synergies, but in less collaborative or open settings, it could have also risked duplication of efforts.

## Conclusions

Our short report highlights the added value of adapting the model of diffusion of innovations for understanding barriers and facilitators to implementing health systems interventions, such as the ones implemented by the MANEST and MANIFEST projects. Implementing interventions through a PAR approach facilitates stakeholder engagement and feeding back of monitoring and evaluation information throughout the implementation period. Furthermore, this approach facilitated the support for strong local leadership through both dissemination and active decision-making about the project, building on the relationships that the teams had developed locally over many years. Designing interventions to support existing processes enhance the likelihood that they will be compatible with the system, though entrenched social norms and customs at the community level need to be understood and appreciated early in the process as they might pose barriers to future adoption and diffusion. Health systems research projects would benefit from analyses beyond the implementation period, in order to better understand how adoption and diffusion happen, or not, over time, after the external catalyst departs. Finally, blending innovations and implementation research adds value and further reflection on the frameworks, tools, and processes needed to facilitate the synthesis of findings and their feedback into decision-making around scaling up key health interventions would be useful.

## Additional file

Additional file 1: Annex 1. Reflection meeting discussion guide (DOCX 18 kb)

## Data Availability

Data sharing is not applicable to this article as no datasets were generated or analyzed during the current study.

## Abbreviations

DFID: Department for international development
FHS: Future health systems research partner consortium
MakSPH: Makerere university school of public health
MANEST: Maternal and newborn care practices study
MANIFEST: Maternal and neonatal implementation for equitable systems
PAR: Participatory action research
UNEST: Uganda newborn study
VHT: Village health teams
WHO: World Health Organization
