# Supplementary material for: Applying the model of diffusion of innovations to understand facilitators for the implementation of maternal and neonatal health programmes in rural Uganda
Source: Global Health. 2019 Jun 13;15:38. doi: 10.1186/s12992-019-0483-9 (PMC6567581; doi:10.1186/s12992-019-0483-9)
Supplement: Supplementary file 1 — Annex 1. Reflection meeting discussion guide (DOCX 18 kb) [file 12992_2019_483_MOESM1_ESM.docx]

# Annex 1: Reflection meeting discussion guide

1. System readiness and system antecedents: What were some of the facilitators and barriers to launching this innovation and sustaining implementation over the years?
2. Communication and influence: What was the process of reflection and generation of new ideas – within the research team and also with other stakeholders?
   1. How were good ideas identified?
   2. Through what process were they deliberated?
   3. Through what process were they implemented?
3. Diffusion: The two project teams shared team members – was there any diffusion from one project to the other?
   1. If so, how did this happen?
   2. What were some facilitators and barriers to cross-project collaboration and diffusion?
      1. Probe here also about the system antecedents, readiness, overall context
4. Diffusion: In thinking about the geographic diffusion of the project, what was your experience with the diffusion of the intervention or intervention elements to:
   1. Adjacent localities
   2. Localities further away?
   3. Other implementing actors, such as NGOs?
   4. What were some facilitators and barriers to cross-project collaboration and diffusion?
      1. Probe here also about the system antecedents, readiness, overall context
5. Adoption: In your experience, how did various stakeholders adopt the innovation?
6. Adoption: Which aspects of the innovation were most readily adopted? Which ones did you face barriers for?
7. Dissemination: What role did dissemination play in the diffusion process?
8. What were some of the key lessons learned about these topics?
